# Computational signatures of exertion and rest underlie moment-to-moment dynamics of subjective perceptions of effort and fatigue

**DOI:** 10.3758/s13415-026-01417-1

**Published:** 2026-03-09

**Authors:** Tanja Müller, Joseph Milton, Masud Husain, Matthew A. J. Apps

**Affiliations:** 1 Department of Experimental Psychology, University of Oxford, Oxford, UK; 2 Wellcome Centre for Integrative Neuroimaging, University of Oxford, Oxford, UK; 3 Zurich Center for Neuroeconomics, Department of Economics, University of Zurich, Zurich, Switzerland; 4 Nuffield Department of Clinical Neurosciences, University of Oxford, Oxford, UK; 5 Centre for Human Brain Health, School of Psychology, University of Birmingham, Birmingham, UK; 6 Institute for Mental Health, School of Psychology, University of Birmingham, Birmingham, UK

**Keywords:** Physical exertion, Effort, Fatigue, Motivation, Reward, Computational model

## Abstract

Everyday we perform tasks that make us feel fatigued. Theoretical accounts predict that fatigue not only develops due to exertion of effort but also increases how effortful the same action will feel subsequently. However, to date there has been no formalised computational account of subjective perceptions of fatigue and effort, with few studies measuring these sensations directly or quantifying their moment-to-moment changes. In this study, across three experiments, participants were required to exert different levels of physical effort (grip force below maximum capacity) to obtain rewards, rating on each trial how effortful they found exerting force (Experiment 1) or how fatigued they felt (Experiments 2 and 3). Across studies, ratings of fatigue and perception of effort increased over time but also fluctuated on a trial-by-trial basis as a function of both effort exerted and rest. A computational model of fatigue, comprising a recoverable component (with fatigue reducing during rest) and an unrecoverable one (in which it only increases through effort exerted) successfully accounted for subjective responses. It was best able to explain momentary changes in both fatigue and effort perception. This computational model provides insights into the brain mechanisms underpinning the close, dynamic relationship between sensations of fatigue and effort.

**Supplementary Information:**

The online version contains supplementary material available at 10.3758/s13415-026-01417-1.

## Introduction

People perform sustained effortful tasks on a regular basis everyday. However, our performance and the motivation to persist in demanding tasks typically decline over time, in terms of both speed and accuracy of the required cognitive operations and physical acts (Boksem et al., 2006; Carroll et al., 2017; Enoka et al., 2011; Helton & Russell, 2015; Inzlicht et al., 2014; Mackworth, 1964; Matthews et al., 2023; Meyniel et al., 2013; 2014; Müller et al., 2021; Pageaux & Lepers, 2016; Sidhu et al., 2013; Tanaka et al., 2014; Tanaka & Watanabe, 2012; Vøllestad, 1997; Warm et al., 2008). Such changes in performance are often attributed to sensations of fatigue. Neurocognitive theories propose overlapping neural correlates of effort and fatigue and suggest that as fatigue heightens, it leads to increases in perceptions of how effortful an ongoing task is, in turn leading to reductions in performance (Boksem et al., 2006; Boksem & Tops, 2008; Müller & Apps, 2019). However, few studies systematically measure people’s subjective perceptions of how effortful a task is, or how fatigued they feel, despite such claims being central to a large proportion of research examining the effects of fatigue on behaviour (Halperin & Vigotsky, 2024). When such measures are taken, they are usually interspersed across multiple trials or blocks of trials, requiring repeated exertions of different actions or cognitive operations. As a result, the brain mechanisms and computations that underlie how subjective perceptions of fatigue, and effort, change from moment to moment are poorly understood.

Previous research has suggested that the rate of decline in performance, and thus putatively changes in levels of fatigue and the perception of effort, depend on the demands of the task being performed (Jones & Hunter, 1983; Müller & Apps, 2019; Stevens & Cain, 1970; Taylor & Gandevia, 2008). Specifically, the more demanding a task is and the more effort it requires to be executed successfully, the greater levels of fatigue will be, leading to the same difficulty of a task to be perceived as more effortful (Hockey, 2011). Yet, changes in performance do not only decline over time. Breaks in tasks can prove restorative to performance (Helton & Russell, 2015; Meyniel et al., 2014; Müller et al., 2021). Additionally, even after an extended, demanding task has significantly reduced performance, offering larger rewards for performance can still improve performance (Boksem et al., 2006), with some evidence that rewards can reduce perceptions of effort or fatigue in some cases (Dobryakova et al., 2018; 2020; Pooresmaeili et al., 2015). Thus, theories suggest that fatigue changes due to the effort exerted, whether rests are taken, and the rewards that can be obtained.

While the arguments for these putative features of fatigue and perception of effort are compelling, the evidence supporting them is limited. Specifically, most studies only examine changes in performance or decision-making, not subjective reports of how effort and fatigue change (Asplund & Chee, 2013; Boksem et al., 2006; Boksem & Tops, 2008; Lorist et al., 2005; Meyniel et al., 2013; 2014; Meyniel & Pessiglione, 2014; Müller & Apps, 2019; Soutschek & Tobler, 2020). Moreover, when they do take such measurements, they are usually only taken after extended periods of time, often in a pre- versus post-fatiguing task design or across blocks of a task (Hogan et al., 2020; Marcora, 2009; Marcora et al., 2009; Milyavskaya et al., 2019; Van Cutsem et al., 2017). Typically, they do not specifically examine trial-by-trial changes in effort or fatigue perception (de Morree et al., 2014; Dobryakova et al., 2018; 2020; Dora et al., 2021; Hopstaken et al., 2015; Möckel et al., 2015; Parry et al., 2011; Pooresmaeili et al., 2015; Rollwage et al., 2020). As a result, the moment-to-moment dynamics of both fatigue and effort perception are obscure.

This is particularly problematic for existing theoretical accounts. If changes in fatigue, effort perception, and motivation are closely interlinked and dynamically changing from moment-to-moment (Matthews et al., 2023; Müller et al., 2021; Müller & Apps, 2019), people’s ratings of fatigue and effort perception at the end of a block may be confounded by them changing how much effort they invest at different points during that block. Therefore, taking infrequent “snapshot” measurements may miss many facets for how fatigue and effort develop.

Recently, some investigations have provided insight into how fatigue might impact motivation dynamically on a moment-to-moment basis (Matthews et al., 2023; Meyniel et al., 2013; 2014; Meyniel & Pessiglione, 2014; Müller et al., 2021; Müller & Apps, 2019). Using experimental designs in which participants make choices about whether to exert different levels of physical effort (e.g., grip force) for different magnitudes of reward, it has been shown that motivation fluctuates during a task with two different components that putatively reflect fatigue (Matthews et al., 2023; Müller et al., 2021). First, there is a recoverable component, in which fatigue increases as a function of how much effort is exerted, but decreases during periods of rest. Second, there is an unrecoverable component that gradually increases through effortful exertion but does not decline with short periods of rest.

These dynamic fluctuations in fatigue have been captured using a computational modelling-based approach that predicts changes in decisions on a trial-to-trial basis. This approach is advantageous. It can parameterise both how sensitive each person is to the different components that lead to a build-up of fatigue. It has also been used in combination with neuroimaging to probe brain mechanisms underlying how the motivation to exert effort is impacted by fatigue, indicating that neural activity in frontostriatal systems fluctuates with fatigue during decision-making (Müller et al., 2021). However, similar computational approaches have not been used thus far to investigate perceptions of effort. Thus, it is unclear whether trial-to-trial changes in perceptions of effort are impacted by both the recoverable and unrecoverable components of fatigue. Moreover, while there is initial evidence (Matthews et al., 2023; Müller et al., 2021), it remains to be confirmed whether sensations of fatigue can be explained by these models and to be established if perceived effort and fatigue are impacted by rewards as previous theories based on performance might suggest (Boksem et al., 2006; Boksem & Tops, 2008; Hockey, 2011; Pooresmaeili et al., 2015; Rollwage et al., 2020).

**Fig. 1:**
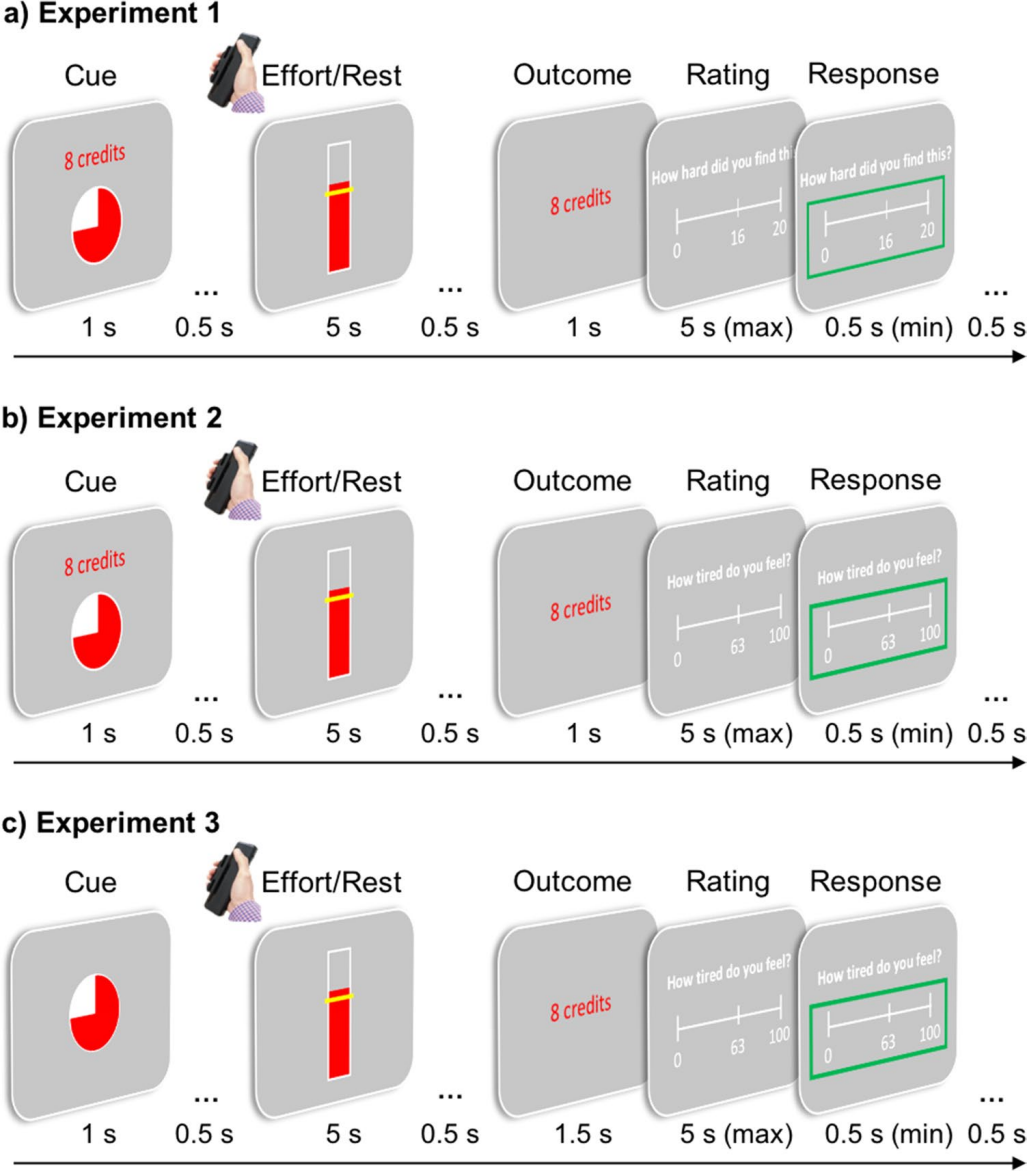
Task structures. Each experiment began with a calibration phase which estimated a participant’s maximum voluntary contraction (MVC), and a training phase where participants became familiar with the levels of effort they would execute in the main tasks (0, 30%, 39%, 48% MVC). In the main tasks, participants were required to squeeze to one of these three levels for 3 s out of a 5-s window, or rest for 5 s, to obtain rewards. On each trial, they were required to execute the required force to the required level (indicated by a red fill moving up a white bar, with the force required indicated by a yellow line). If they successfully maintained that force for 3 s across the window, they then received a reward in the outcome phase; if not, they were given 0 credits. Lastly, in each experiment, they provided a rating on a visual analogue scale. In Experiment 1, **(a)** participants saw the amount of force required before each trial, as well as the amount of credits they would receive if successful (6, 8, or 10 credits), with higher credits increasing their bonus payment. At the end of every trial, they then rated between 0 and 20 how hard (effortful) they felt the trial was. There were 120 trials with the only breaks being the 5 s trials of “rest.” Experiment 2 **(b)** was almost identically structured but differed in that the rating participants provided (between 0 and 100) was for how tired (fatigued) they felt at that point. Thus, these experiments could measure fluctuations in the perception of effort over trials or fatigue. Experiment 3**(c)** differed in that participants were not informed of the credits that they would receive on each trial prior to exerting the force, removing the possibility of participants squeezing harder for higher rewards.

We developed three tasks to systematically examine how sensations of effort and fatigue develop over repeated physical exertions as a function of effort exerted, rest taken, and rewards obtained (Fig. 1). On every trial, participants were required to exert a specified amount of effort (grip force) for a certain amount of reward and afterwards rated on a visual analogue scale how hard (effortful) they found it (Experiment 1) or how tired they felt (Experiments 2 and 3). While in Experiments 1 and 2, the reward on each trial was presented to participants *before* working or resting, in Experiment 3 information on the reward associated with that trial was only presented to participants *after* they had worked or rested. This allowed testing for any effect of reward crucially without potential confounds of behaviour (such as participants exerting more force when higher rewards are at stake) (Bonnelle et al., 2015; 2016; Oudiette et al., 2019).

Using this design, we tested the hypothesis that the perception of effort and sensations of fatigue increase depending on the amount of physical effort exerted, but also decrease as a function of time rested. More specifically, we predicted that a computational model of fatigue characterised by recoverable (RF) and unrecoverable (UF) components would be best to characterise ratings of effort and fatigue.

## Methods

### Participants

Participants were recruited through the Oxford Psychology Research participant recruitment scheme and online bulletin boards, typically comprising university students and staff as well as members of the public in the vicinity of Oxford. Participants were informed that the study would involve squeezing a hand-held device. The sample size was based on related previous studies assessing trial-to-trial fluctuations in self-reported fatigue or in the subjective evaluation of effort costs (Müller et al., 2021; 2022). Pre-established exclusion criteria included a history of neurological or psychiatric illness. Forty-two individuals participated in Experiment 1, of which one reported psychiatric illness and was excluded from the analyses. The final sample of 41 participants who did not report any history of neurological or psychiatric illness comprised 21 females and had a mean age of 23.93 years (standard deviation [*SD*] = 5.18; range 18–40). In Experiment 2, a new sample consisted of 40 participants (24 females) with a mean age of 24.18 years (*SD* = 4.68; range 19–35) and with no history of neurological or psychiatric illness. For Experiment 3, a new sample of 41 individuals participated, of which one reported psychiatric illness and was therefore excluded from the analyses. The final sample of 40 participants, which we have partly analysed previously (Müller et al., 2021), comprised 24 females and had a mean age of 25.53 years (*SD* = 5.63; range 18–40). The research was approved by the South Central – Oxford C Research Ethics Committee (14/SC/0044) and the South Central – Oxford A Research Ethics Committee (18/SC/0448), and written informed consent was obtained from all participants prior to the experiment in accordance with the ethical standards laid down in the Code of Ethics of the World Medical Association (Declaration of Helsinki).

### Apparatus

The experiments were conducted in a laboratory room with only the participant and the researcher being present. The researcher ensured that the dynamometer was held correctly in the participant’s dominant hand during the experiment. Stimuli presentation and response collection were implemented using custom code in Matlab 2012a (The MathWorks, Inc., USA) and Psychophysics Toolbox extensions (Brainard, 1997), controlled by a PC running the Windows operating system. To examine effects of effort and reward on perceived effort and on perceived fatigue on a trial-by-trial basis, we developed tasks in which effort was operationalised as the amount of force exerted on a handheld dynamometer (TSD121B-MRI; BIOPAC Systems, Inc., USA). This allowed us to systematically set different, individualised effort levels. To reduce potential discomfort, the dynamometer was padded with “squash” tape.

### Experimental design and procedure

Each experiment consisted of three parts: 1) a *Calibration* phase to account for individual differences in grip strength, which was completed before the experiment was explained in full to the participants; 2) a *Training* phase in which participants familiarised themselves with the effort levels used in the main task; and 3) the *Main task*. In the Main task (Fig. 1), participants were asked on every trial to rest or to exert force for rewards (credits). The use of low-to-intermediate effort levels ensured that participants were able to successfully complete this task and that potential effects of outcome uncertainty were mitigated. Subsequently, participants were asked to rate on a visual analogue scale how effortful they found the trial (Experiment 1) or how fatigued they felt (Experiments 2 and 3). Participants were instructed to collect as many credits as they could throughout the experiment, with the total number of credits collected across the task determining their payment. That is, participants were paid £8 for their time and received a bonus payment of up to £4, which was proportional to the credits that they had earnt in the task.

During *Calibration*, each participant’s MVC was measured by squeezing a hand-held dynamometer on three consecutive trials with their dominant hand. Participants were required to apply as much force as possible on each trial, and they received strong verbal encouragement while squeezing. During each attempt, a bar presented on the screen provided feedback of the force being generated. In the second and third attempts, a benchmark (yellow line that represented 105% or 110% of the previous best attempt) was used to additionally encourage the participants to improve their score, with participants instructed to try and reach the yellow line whilst applying as much force as possible. The maximum level of force generated throughout the three attempts was used as the participant’s MVC.

In the *Training* phase, participants practiced reaching each of four effort levels (0, 30%, 39%, and 48% of each participant’s MVC). These effort levels were based on previous work, in which they had generally been achievable over periods of repeated exertion (Matthews et al., 2023; Müller et al., 2021). The trial was successful only when the force generated by the participant exceeded the required level for a sum total of at least 3 s in a 5-s window. Each trial commenced with a cue in the form of a pie chart; the number of red segments indicated the upcoming effort level. During the exertion period, real-time feedback on a participant’s force as well as the target effort level were provided on the screen. To make sure that participants carefully and successfully completed this training, they were awarded one credit for each successful squeeze, while they received zero credits for a failure. In an additional four trials, participants practiced manipulating the rating scale before they completed four full practice trials consisting of the different effort levels and a rating (see Main task) in order to familiarise themselves with the task.

The *Main task* consisted of 120 trials, each requiring participants to either rest or work for credits. In Experiment 1 (Fig. 1a) and Experiment 2 (Fig. 1b), work trials consisted of one of three different effort levels, represented by two to four filled segments in a pie chart (cue) that corresponded to 30%, 39%, and 48% of each participant’s MVC, and one of three different reward levels as numerically displayed above the pie chart (6, 8, or 10 credits). Rest trials were indicated by one filled segment in a pie chart and the number of credits (6, 8, or 10) numerically displayed above it. Experiment 3 (Fig. 1c) was identical to Experiment 2, except for the fact that the cue only indicated the upcoming effort level but not the reward level. In Experiment 3, rewards were presented for 1.5 s and only shown to the participants after they had worked or rested on that trial. In all experiments, effort and reward levels were varied independently and presented in a pseudo-random order to ensure that the repetitions of each effort/reward combination were distributed evenly across the task. Each participant was presented with the same sequence to ensure that any potential differences in behaviour could be attributed to individual characteristics.

After this cue, representing the upcoming effort and—in Experiments 1 and 2—reward levels, participants were required to rest or to exert the respective force on the dynamometer for at least 3 of 5 s to receive the credits. For this purpose, they were presented with a vertical bar that provided them with real-time feedback on their force. The target effort level was indicated by a yellow line superimposed on the bar. If participants had to rest on that trial, the bar was presented for the same duration but with the yellow line displayed at the bottom of the bar. Following this, participants were shown the credits that they had obtained dependent on their success or failure on that trial.

In Experiment 1, participants then were instructed to answer the question, “How hard did you find this?” on a scale ranging from 0 to 20, with 0 representing *no effort at all* and 20 representing *maximum effort*. The starting value on the scale was randomised across the task but identical across participants. They had a maximum of 5 s to confirm or change this value. In Experiments 2 and 3, participants were asked to indicate how tired they feel on a scale ranging from 0 to 100, with 0 representing *not tired at all* and 100 representing *completely exhausted*. Immediately before the first trial, participants were given as much time as they needed to indicate how tired they currently felt (baseline rating). On each subsequent trial, the starting value on the scale was the value the participant had entered on the previous trial, and participants had a maximum of 5 s to confirm or change this value.

Participants could change the value on the rating scales in increments of one by using the left and right arrow keys on a keyboard. They confirmed their chosen value by pressing the downward arrow key, which was indicated by a green frame appearing around the rating scale. To ensure that participants reported their feelings accurately, they were instructed that they could increase or decrease their effort or fatigue ratings or could keep them constant, that they should respond honestly, and that their ratings would neither affect the remainder nor the outcome of the experiment in any way.

### Statistical analysis

To examine how people’s sensations of effort and fatigue changed on a trial-to-trial basis, we first analysed participants’ effort ratings on trial *n* (Experiment 1) and changes in participants’ fatigue ratings from trial *n*−1 to trial *n* (Experiments 2 and 3) with linear mixed-effects models (LMM) using the *lmer* function from the lme4 package (Bates et al., 2015) in R 3.5.2 (R Core Team, 2018) with the maximum likelihood estimation method. In addition, we examined their behaviour (force produced) on trial *n* (all experiments) using similar models. Following previous work (Lockwood et al., 2017), force on each trial was calculated as the area under the curve (AUC) of the voluntary contraction trace recorded from the dynamometer, using the *trapz* function in Matlab. For every participant, the area under the curve on any given trial was normalised by the maximum value calculated for this participant to account for interindividual differences in force exerted.

Trials *n* in which participants worked and trials *n* in which they rested were examined in separate LMMs. This was based on the assumption that perceived effort and changes in perceived fatigue would be (linearly) scaled by the amount of effort when they exerted effort, while taking rests might have a differential impact on the perception of effort and fatigue. Only trials *n* in which participants had successfully reached the effort level for the required duration and thus obtained the credits were included in the models. Overall, this resulted in the exclusion of *M* = 3.07% (*SD* = 6.76) trials in Experiment 1, *M* = 1.81% (*SD* = 4.51) trials in Experiment 2, and *M* = 4.21% (*SD* = 6.60) trials in Experiment 3. Unsuccessful trials were not further analysed because of their rare occurrence.

In all models, a subject-level random intercept was included which allowed modelling of potential variability between participants. When the additional inclusion of a random slope on the main effect of effort or the main effect of cumulative effort and their interaction resulted in better model fits, in terms of the Akaike Information Criterion (AIC) (Akaike, 1974) and the Bayesian Information Criterion (BIC) (Schwarz, 1978), with a different pattern of results, we note this in the respective results section. Dependent variables and predictors were coded as continuous variables, with predictors being z-scored relative to the mean and standard deviation for each sample. Effects were tested for statistical significance using a Type II Wald chi-square test, i.e., χ^2^ and *p*-values refer to comparisons between the tested model and the same model without the respective main effect or interaction of interest. Coefficients and standardised coefficients with 95% confidence intervals were derived from the linear mixed-effects models using the *model_parameters* function from the parameters package (Lüdecke et al., 2020) and are reported in Supplementary Tables 1 to 11.

### Computational modelling

**Fig. 2:**
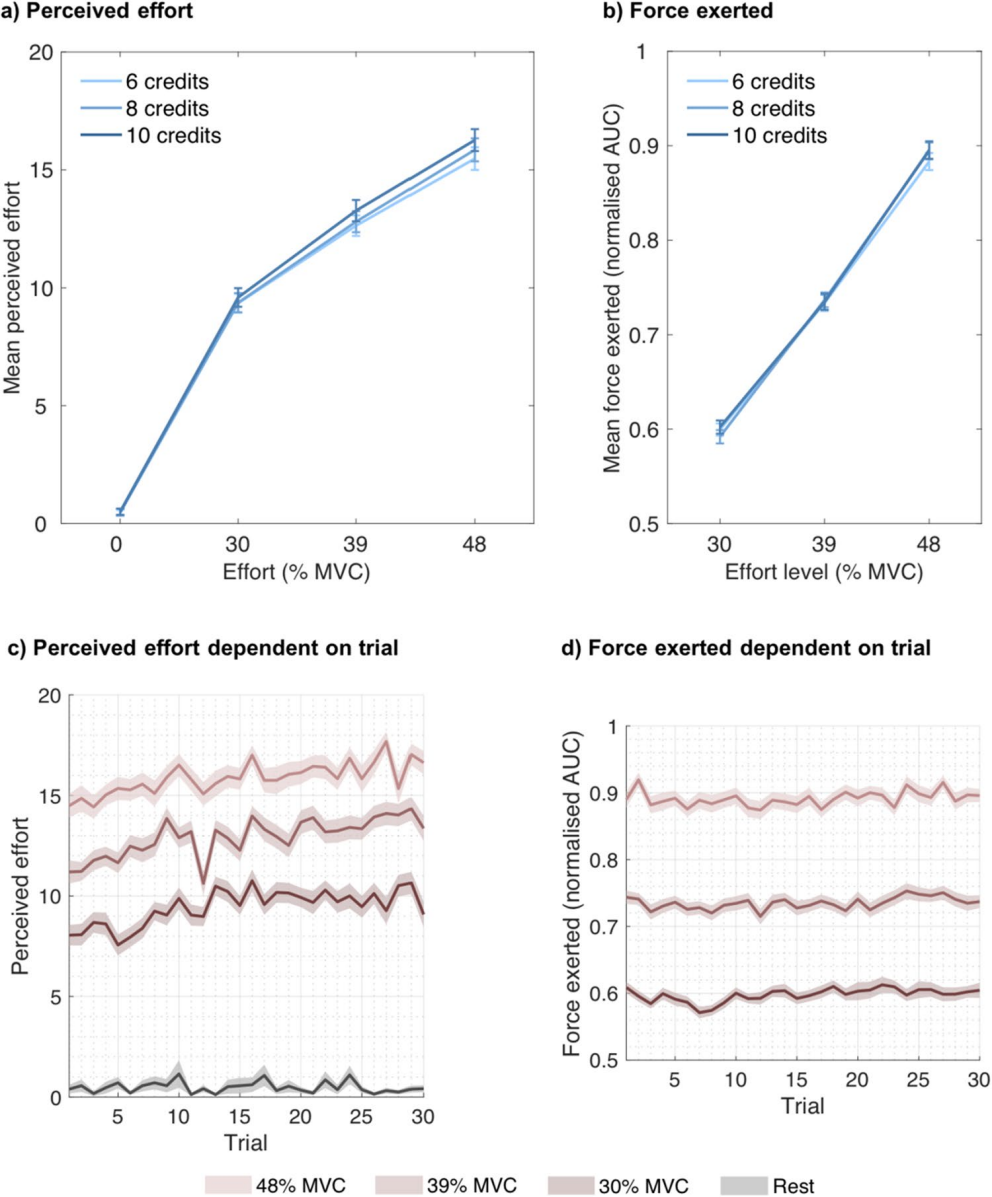
**Perceived effort and produced force as functions of effort, reward and trial number. (a)** Mean ratings on the question, “How hard did you find this?” as a function of rest (0% MVC), effort level and reward (credits earned), showing that the perception of effort increases with exertion and that higher rewards are associated with slightly higher perceived effort. **(b)** Mean force exerted on a trial, calculated as the area under the curve (AUC) and normalised for each participant, dependent on effort level and rewards presented on that trial. **(c)** Participants’ mean ratings on the question, “How hard did you find this?” as a function of rest (0% MVC, black line), effort level, and the trial in which the respective rest/effort level was required throughout the task. Analyses show that ratings of perceived effort increase across trials. **(d)** Mean force exerted on a trial, calculated as AUC and normalised for each participant, as a function of effort level and the trial in which the respective effort level was required throughout the task. Only successful trials in which participants exerted the required amount of force and earned the credits associated with that trial were included. Error bars and shaded areas represent standard errors of the means.

To more specifically predict and quantify the effects of exertion and rest on perceived effort and fatigue on a trial-by-trial basis, we fitted different computational models to each participant’s effort or fatigue ratings in Experiments 1 to 3. To account for the observed effects of reward on force and for dynamics in force production, we included the actual force produced (area under the curve, calculated as described in the previous section and multiplied by 10) and not simply the requested effort level in these models. Because only three, low-to-moderate effort levels were used in our experiments, in addition to rest trials, effects of effort were modelled in a linear fashion (Figs. 2 and 5).

Our full fatigue model assumed that fatigue would increase with exertion and would be partially recoverable and decrease with time spent resting but would also have a gradually increasing unrecoverable component, which does not recover with the rest taken on a trial (Matthews et al., 2023; Müller et al., 2021). In this model, fatigue ($F$) on each trial ($t$) was calculated as the sum of a participant’s baseline fatigue (${F}_{\mathrm{start}}$), recoverable fatigue ($RF$), and unrecoverable fatigue ($UF$):

1 $${F}_{(t)}={F}_{\mathrm{start}}+{RF}_{(t)}+{UF}_{(t)}$$

$RF$ increases dependent on the force exerted ($E$) on a trial (Equation 2) and decreases dependent on the time rested (${T}_{\mathrm{rest}}$) on a trial (here equal to 7.5 in rest trials and 2.5 in work trials; Equation 3):

2 $${RF}_{(t)}={RF}_{(t-1)}+(\alpha * {E}_{\left(t\right)})$$

3 $${RF}_{(t)}={RF}_{(t-1)}-(\delta * {T}_{\mathrm{rest}\left(t\right)})$$

Individuals differ in the degree to which effort increases their fatigue, as reflected by the subject-specific parameter $\alpha$, and in how quickly they recover during rest, reflected by the parameter $\delta$. Unlike RF, $UF$ accumulates depending on the effort exerted across the whole task and is not restored by resting during a trial (Equation 4). The parameter $\theta$ thus represents how much different individuals are susceptible to the build-up of fatigue that cannot be easily recovered:

4 $${UF}_{(t)}={UF}_{(t-1)}+(\theta * {E}_{(t)})$$

With regard to Experiment 1, these fatigue terms were integrated into the modelling of subjectively perceived effort ($$PE)$$ on each trial (Equation 5). For rest trials, PE was assumed to be 0, following theoretical assumptions and our empirical data (Fig. 2c). For work trials, PE was dependent on $$E$$ on a trial, weighted by a subject-specific parameter $$\gamma$$ that captures interindividual differences in effort perception as well as by the estimated fatigue levels:

5 $${PE}_{(t)}=(\gamma +{F}_{\left(t\right)})*{E}_{(t)}$$

Initial RF and UF values were set to 0, with RF and UF subsequently updated on each trial according to the respective model and added to the fatigue level indicated by the respective participant before the start of the Main task (baseline rating, or 0.01 if the baseline rating was 0 (Experiments 2 and 3) or if no baseline rating was collected (Experiment 1)). Based on theoretical considerations, only parameter values (α, δ, θ, $\gamma$) ≥ 0 and RF estimates ≥ 0 were allowed.

The fit between the model and the data, as indexed by the sum of squared residuals (RSS) between the participant’s ratings and the model’s estimates, was optimised by using *fminsearch* function in Matlab 2017b (The MathWorks, Inc., USA), i.e., model parameters were changed to minimise the difference between each participant’s actual rating and the rating predicted by the model for each trial. To maximise the chances of finding global rather than local minima, parameter estimation for the full model and for all alternative models was repeated over a grid of initialisation values, with six initialisations (ranging from 0 to 1) per parameter. The optimal set of parameters for each model was used for model comparison.

To verify whether the parameters used to quantify the effects of effort and rest on fatigue were necessary, alternative fatigue models were fitted to participants’ ratings. This included models in which there was an effect of UF only (i.e., $\theta$ being fitted) or an effect of RF only (i.e., $\alpha$ and $\delta$ being fitted). In addition, two further mathematically plausible but theoretically unlikely models were included, which used only one parameter to scale the effect of effort and rest on recoverable fatigue (i.e., only $\alpha$ being fitted across both work and rest trials). In one of these models, fatigue was only comprised by this one parameter for RF, whereas in a second model, fatigue comprised UF plus the one parameter for RF. In addition, for the modelling of effort ratings in Experiment 1, we tested one null model that captured interindividual differences in effort perception without any effect of fatigue over the course of the task (i.e., only $\gamma$ being fitted).

To investigate the models’ relative abilities to predict the rating data, the model fits were compared by using the AIC, with lower values indicating better fit. The AIC was calculated according to the following formula, with $n$ representing the number of observations, i.e., the number of trials, and $d$ representing the number of parameters:

6 $$\mathrm{AIC}=n*\mathrm{ln}(\frac{\mathrm{RSS}}{n})+2*d$$

In addition, to test whether the full model had the highest probability of being the most frequently best fitting model to participants’ ratings, we calculated exceedance probabilities for each model (Stephan et al., 2009) based on inverted AICs using the *spm_BMS* function from SPM12 (https://www.fil.ion.ucl.ac.uk/spm). Reported *R*^2^ values represent squared correlation coefficients between observed and predicted data.

## Results

### Experiment 1

Participants performed an effort rating task where on each trial they were required to perform a level of physical effort at a percentage of their own maximal voluntary contraction (MVC). On each trial they first saw a cue indicating the level of force required (30%, 39%, or 48% of their MVC, or a 0% rest) and the reward (6, 8, or 10 credits that increased a bonus payment) that they could obtain if they successfully maintained that force for 3 s. Following this they were required to exert that force for 3 s out of a 5-s window. Participants then obtained the outcome indicating whether they received the reward or not. Lastly, they rated how effortful that force was between 0 and 20.

#### Perceived effort depends on current and previous levels of exertion

First, we wanted to test statistically whether the perception of effort changed over trials in a manner consistent with fatigue increasing the perception of effort. We performed a mixed-effects model with the z-scored predictors of required effort level on a trial (2, 3, or 4), offered reward on a trial (6, 8, or 10 credits), cumulative effort (sum total of effort levels (levels 2, 3, or 4) during the task prior to the current trial) and their interactions, including subject as a random effect, on successfully completed work trials (95.91% of those trials were successfully completed on average). As would be predicted based on previous theories of fatigue and effort perception, effort, reward, as well as their interaction predicted effort ratings (effort: χ^2^(1) = 4,195.950, *p* <.001; reward: χ^2^(1) = 22.112, *p* <.001; effort × reward: χ^2^(1) = 4.472, *p* =.034). Specifically, the higher effort levels were associated with higher ratings of effort, as were higher rewards (Fig. 2a). Strikingly, cumulative effort was also a significant predictor of a higher perception of effort (χ^2^(1) = 240.504, *p* <.001), indicating that participants’ perception of effort increased over repeated exertion (Fig. 2c). There was no significant interaction between cumulative effort and any other predictor (cumulative effort × effort: χ^2^(1) = 0.113, *p* =.737; cumulative effort × reward: χ^2^(1) = 2.306, *p* =.129; cumulative effort × effort × reward: χ^2^(1) = 1.460, *p* =.227), indicating that as the amount of effort (and time) accumulated, participants perceived the effort levels as more difficult, across all levels, not specifically at higher or lower levels.

We then separately analysed rest trials. In an LMM with reward, cumulative effort and their interaction as predictors and subject as a random effect, neither reward (χ^2^(1) = 0.152, *p* =.697) nor cumulative effort (χ^2^(1) = 0.173, *p* =.677) nor their interaction (χ^2^(1) = 0.186, *p* =.666) affected effort ratings (Fig. 2). The finding that sensations of effort were negligible on rest trials (Fig. 2) and that there was no evidence that they were related to the amount of reward in these trials suggests that participants were using the rating scale accurately and as expected.

In the analysis above, we found that reward increased perceptions of effort. There are two possible explanations for this: 1) receiving a reward makes participants feel like they worked harder even though the same effort was exerted; or 2) the incentive of a higher reward made participants increase their force exertion, and thus the higher perception of effort was due to an actually increased level of effort even though the level required was the same.

To test this, we predicted force exerted in a mixed model with predictors of effort, reward, and cumulative effort in successfully exerted work trials. Force production systematically varied with the effort levels set in the experiment (χ^2^(1) = 19,629.648, *p* <.001). In addition, higher expected rewards led to increased force (χ^2^(1) = 4.217, *p* =.04), mostly irrespective of the effort level that was required (reward × effort: χ^2^(1) = 3.456, *p* =.063; Fig. 2b). Force also increased over repeated exertion (χ^2^(1) = 19.108, *p* <.001), although this only had a small impact on the force exerted (Fig. 2d). None of the other interactions were significant (cumulative effort × effort: χ^2^(1) = 2.012, *p* =.156; cumulative effort × reward: χ^2^(1) 1.179, *p* =.278; cumulative effort × effort × reward: χ^2^(1) = 1.298, *p* =.255). Crucially, in an alternative LMM with a lower AIC and BIC, including additional random slopes for cumulative effort and effort as well as their interaction, the interaction of effort and reward was significant (χ^2^(1) = 4.586, *p* =.032), but the main effect of cumulative effort was not significant (χ^2^(1) = 3.723, *p* =.054). Overall, these analyses therefore suggest that the effects of reward on perceived effort described above may partly be due to participants exerting more force than required when higher rewards are at stake.

#### Recoverable and unrecoverable components underlie fluctuations in perceived effort

Next, we used computational modelling to test whether a previously developed model of fatigue can account for fluctuations in sensations of effort (Matthews et al., 2023; Müller et al., 2021). We hypothesised that a full model would be the best fit to our ratings. This model contained four parameters comprising: 1) a static difference in how effortful the produced force was ($\gamma )$, 2) a recoverable component with parameters scaling the increased perception of effort over trials due to effort ($\alpha )$, and decreases in effort perception with rest ($\delta$), and 3) an unrecoverable component with a parameter that increases over trials but does not decline with rest ($\theta )$. The model was compared with five alternatives that hypothesised simpler mechanisms leading to an increase in perception of effort, or no change, using AIC and exceedance probability.

**Fig. 3:**
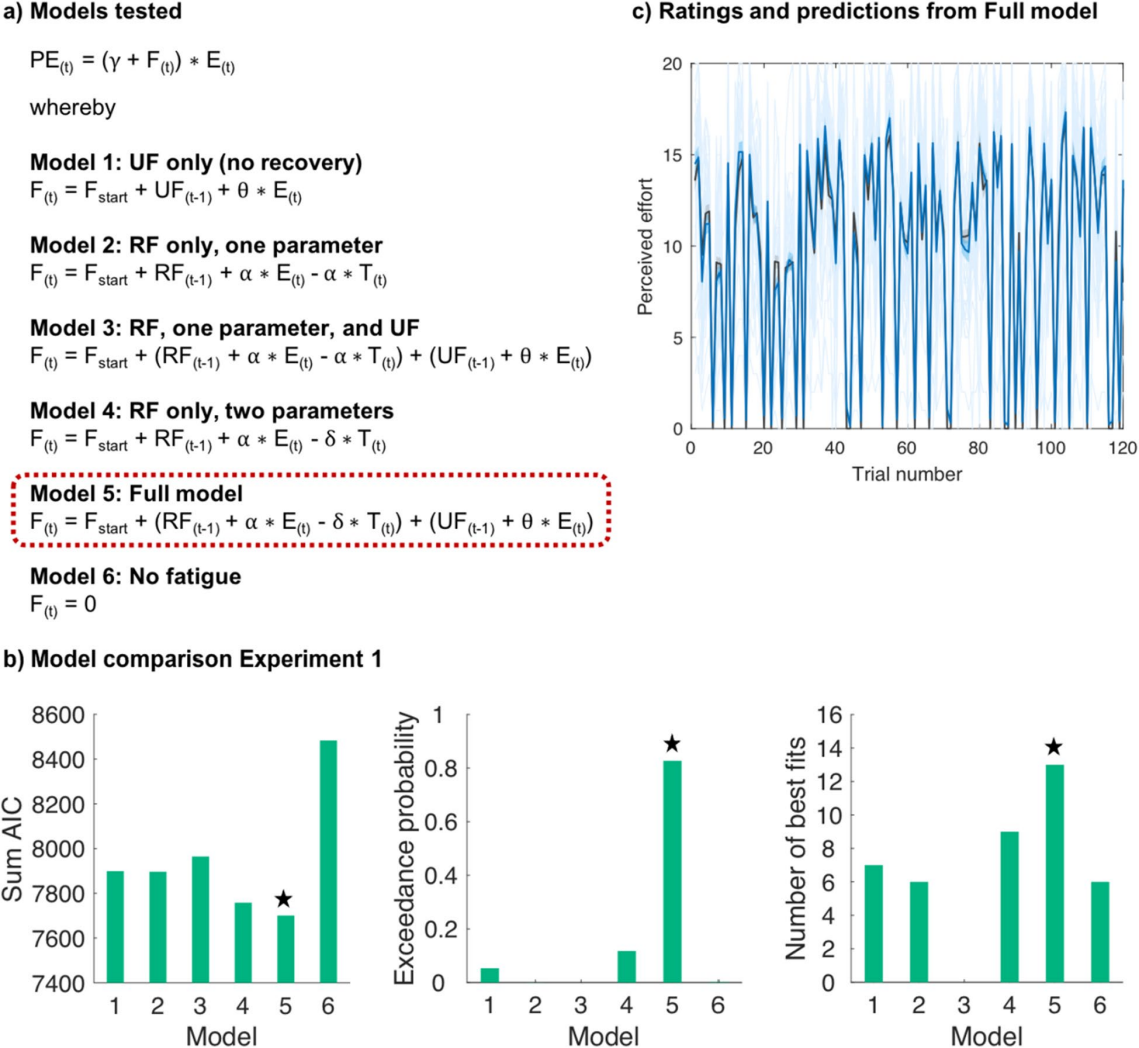
**Modelling perceived effort. (a)** List of models compared. **(b)** Model comparison results for Experiment 1. The x-axis is depicting the model number and the y-axis the sum of the Akaike Information Criterion (AIC) score, the Exceedance probability, or the number of participants for whom ratings were best explained by the respective model according to individual AICs, indicating that participants’ development of perceived effort was overall best described by the Full model (Model 5), as indicated by the star. We note that when the numbers of best fits were calculated based on RSS and not based on AIC, the Full model (Model 5) explained ratings best in 29 participants, whereas Model 4 in this case was the best fit for 12 participants. **(c)** Individual participants’ effort ratings (light blue) and group mean (blue) across the task, alongside mean effort ratings as predicted by the Full model (black). Shaded areas represent standard errors of the means. PE = perceived effort on trial t; F = fatigue on trial t; UF = unrecoverable fatigue; RF = recoverable fatigue; E = force exerted; T = time rested.

**Fig. 4:**
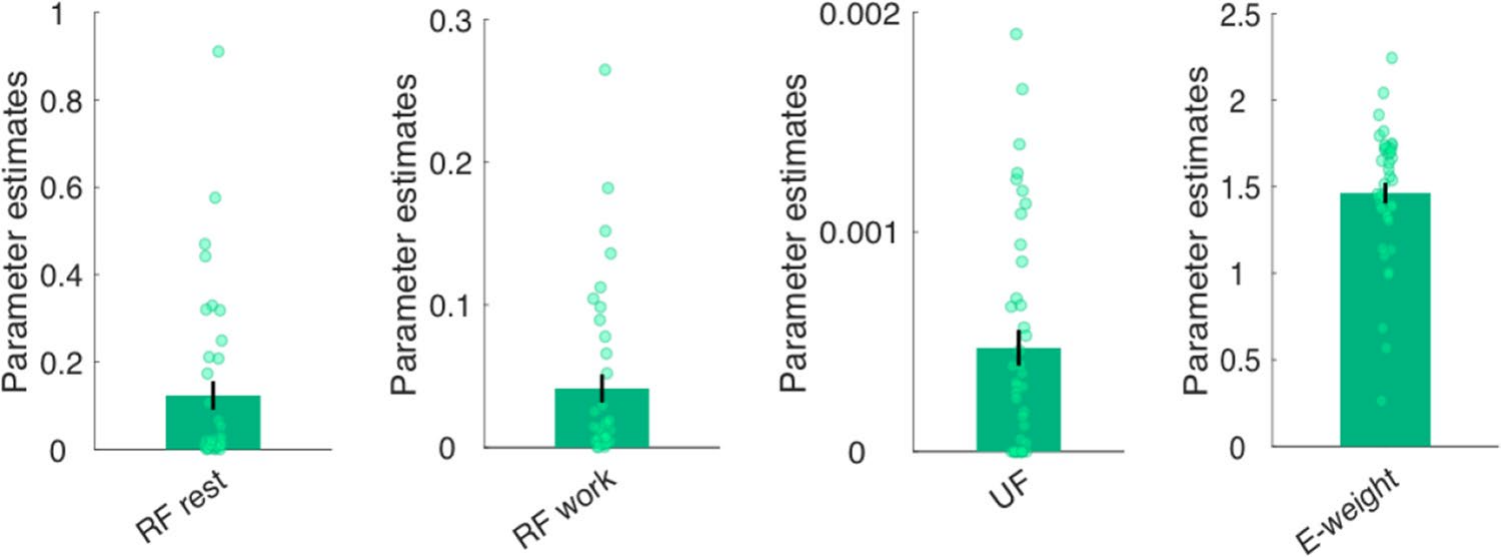
Model parameters from the Full model in Experiment 1. Displayed are parameter estimates for each participant (green dot) and the average across participants for the two recoverable fatigue parameters (RF; *n* = 38), the unrecoverable fatigue parameter (UF; *n* = 41) and the effort weighting parameter (E-weight, *n* = 41). Three participants’ RF parameters were not included for display purposes due to high values. Error bars represent standard error of the mean (SEM).

Analyses revealed that the full model best predicted participants’ perceived effort (Fig. 3). This model had a lower AIC and higher exceedance probability than the alternative models, and it fit the effort ratings well (median *R*^2^ =.88; Fig. 3c). These results support the notion that our model is also able to capture trial-by-trial dynamics in perceived effort. We present parameter estimates for the winning model (Fig. 4) as well as examples of participants with different parameter values (Supplementary Fig. 1a) to illustrate interindividual variability. For completeness, we additionally tested five models in which the fatigue terms were not multiplied with effort but instead simply added to the product of $\gamma$ and effort. However, these models did not fit the data better than our winning model described in Fig. 3 in terms of both AICs and exceedance probabilities.

### Experiments 2 and 3

Experiment 1 revealed that subjective perceptions of effort fluctuate with a putative model of fatigue, suggesting that how effortful something feels depends on how fatigued one feels. However, previously this fatigue model had been deployed in studies in which participants made decisions about whether to exert effort for reward. In the following experiments, we now fitted this model to data in which the design was identical to Experiment 1, except that participants rated their level of fatigue instead of their perception of effort (Experiment 2) and controlled for the possible incentivisation effect of reward on effort (Experiment 3) identified in Experiment 1. Experiment 3 differed from Experiment 2 only in the fact that participants were not informed of the reward on offer before exerting effort or resting. They were given a pseudo-random reward (6, 8, or 10 credits) on each successfully performed trial, and they rated their fatigue after receiving the outcome. Thus, we could examine whether reward had effects on fatigue ratings even if the incentive could not influence force exertion.

#### Perceived fatigue depends on current and previous levels of exertion

First, we examined whether feelings of fatigue increased over the course of the task in our experiments. Indeed, participants’ fatigue ratings on a visual analogue scale ranging from 0 to 100 increased in both Experiments 2 and 3 (Figs. 7d and 7e).

Next, we tested the effects of effort and reward on the change in fatigue from trials *n*−1 to trials *n* in which participants had successfully worked in Experiment 2 (97.58% of those trials were successfully completed on average). Required effort level (2, 3, or 4), offered reward (6, 8, or 10 credits), and the interaction of effort and reward were included as predictors of interest in the mixed-effects model. To be able to directly compare analyses from the different experiments, cumulative effort (sum total of effort levels (levels 2, 3, or 4) during the task prior to the current trial) and its interactions with the other predictors were added to the model. Effort, reward, and cumulative effort were z-scored beforehand, and a random intercept for subject was included.

**Fig. 5:**
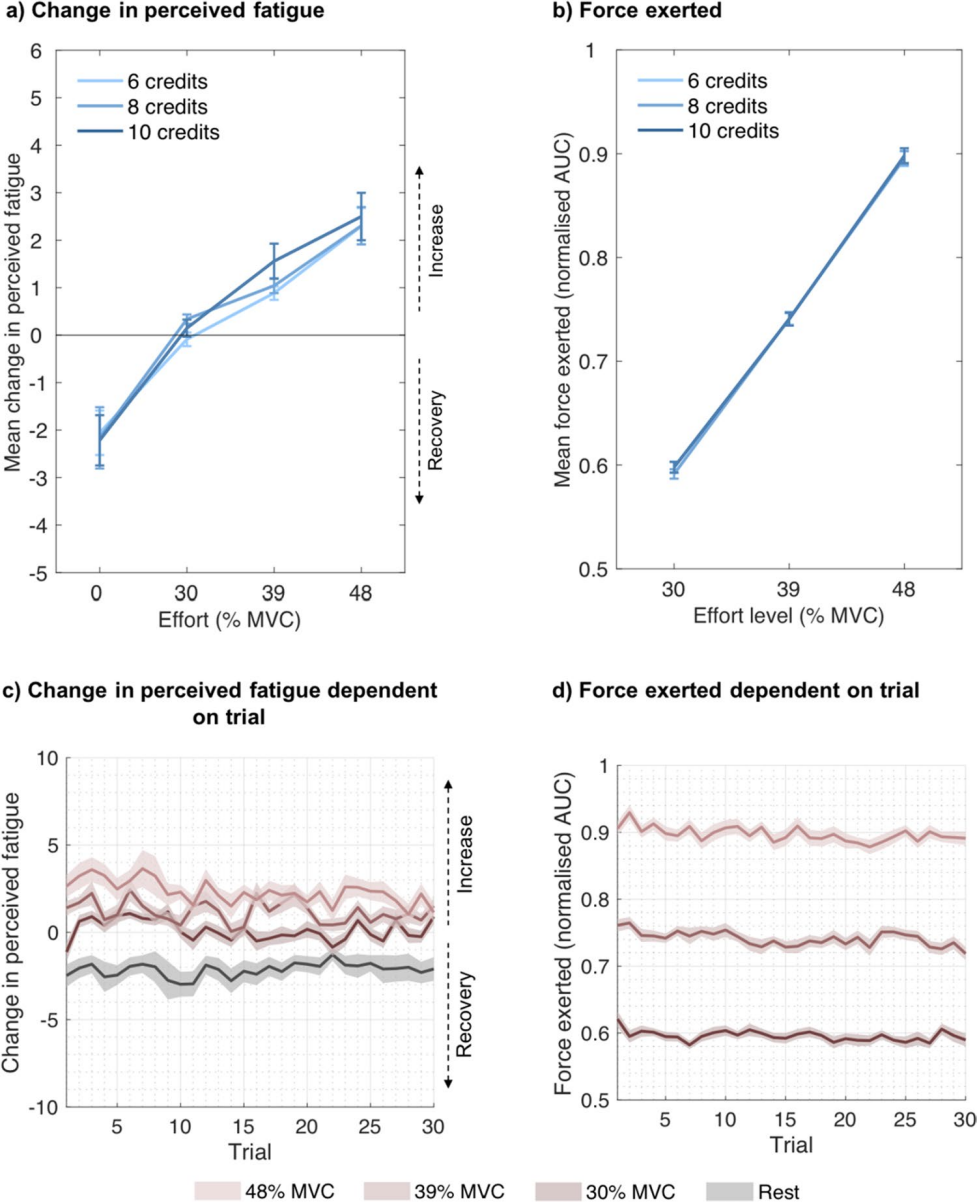
**Change in perceived fatigue and force exerted in Experiment 2 as a function of effort, reward and trial number. (a)** Mean change in participants’ ratings on the question, “How tired do you feel?” from trial *n*−1 to trial *n* as a function of rest (0% MVC), effort level, and reward (credits earned), showing that perceived fatigue decreases with rest and increases with exertion, in which case higher rewards are associated with slightly higher fatigue increase. **(b)** Mean force exerted on a trial, calculated as the area under the curve (AUC) and normalised for each participant, dependent on effort level and rewards presented on that trial. **(c)** Mean change in participants’ ratings on the question, “How tired do you feel?” from trial *n*−1 to trial *n* as a function of rest (0% MVC), effort level, and the trial in which the respective rest/effort level was required throughout the task. **(d)** Mean force exerted on a trial, calculated as AUC and normalised for each participant, as a function of effort level and the trial in which the respective effort level was required throughout the task. Only successful trials in which participants exerted the required amount of force and earned the credits associated with that trial were included. Error bars and shaded areas represent standard errors of the means.

Analyses confirmed that the higher the effort, the higher the increase in fatigue (χ^2^(1) = 287.873, *p* <.001). In addition, higher obtained rewards were associated with a higher increase in fatigue (χ^2^(1) = 11.598, *p* <.001; Fig. 5a), while the interaction of effort and reward was not significant (χ^2^(1) = 0.102, *p* =.75). Furthermore, the change in fatigue decreased over the course of the experiment (χ^2^(1) = 27.104, *p* <.001; cumulative effort × effort: χ^2^(1) = 4.086, *p* =.043; Fig. 5c). None of the other interactions were significant (cumulative effort × reward: χ^2^(1) = 0.609, *p* =.435; cumulative effort × effort × reward: χ^2^(1) = 1.096, *p* =.295).

Analyses of the rest trials in a separate LMM, with reward, cumulative effort, and their interaction as fixed effects, did not reveal a significant effect of reward on the decrease in fatigue from the previous to the current trial when resting (χ^2^(1) = 0.99, *p* =.32), and the effects of rest on fatigue did not significantly change with cumulative effort (χ^2^(1) = 3.422, *p* =.064) nor was there a significant interaction (χ^2^(1) = 0.122, *p* =.727). These results show that in this experiment fatigue changes moment-to-moment, depending on the effort exerted but crucially also on the reward obtained. Higher effort and higher reward both resulted in greater increases in fatigue, yet reward did not affect changes in fatigue during rest.

Next, we wanted to test whether higher reward led to increased force production, which could possibly account for the effects of reward on changes in perceived fatigue, as well as to test whether force declined over repeated exertions, which could possibly account for the effects of cumulative effort on changes in perceived fatigue. To test this, we ran an additional analysis predicting force by effort level, reward, cumulative effort, and all interactions, again only including those work trials in which participants had performed the trial successfully. Effort significantly predicted the force exerted (χ^2^(1) = 20,471.206, *p* <.001), indicating that participants adhered well to the task instructions. While force production indeed overall decreased over the course of the experiment (cumulative effort: χ^2^(1) = 43.746, *p* <.001, cumulative effort × effort: χ^2^(1) = 3.57, *p* =.059; cumulative effort × reward: χ^2^(1) = 2.144, *p* =.143; cumulative effort × effort × reward: χ^2^(1) = 1.978, *p* =.16; Fig. 5d), there was no evidence that higher reward significantly resulted in higher force (reward: χ^2^(1) = 1.668, *p* =.197; reward × effort: χ^2^(1) = 1.638, *p* =.201; Fig. 5b). We note, however, that when random slopes on the main effect of effort and on the main effect of cumulative effort per participant were included in the model, which was associated with a lower AIC and BIC, the interaction of cumulative effort and effort was significant (χ^2^(1) = 8.694, *p* =.003) and the interaction of cumulative effort and reward was close to being significant (χ^2^(1) = 2.771, *p* =.096).

To further test the effects of reward on perceived fatigue, we performed similar analyses to predict changes in fatigue ratings in Experiment 3 in which the reward on each trial was only presented after the force had been produced but before participants rated their fatigue. Therefore, any effect of reward on perceived fatigue would be completely independent of their exerted force, as the incentive for the exertion was only presented after force had been executed. Again, the model included z-scored required effort level (2, 3, or 4), offered reward (6, 8, or 10 credits), cumulative effort, and all interactions as predictors.

**Fig. 6:**
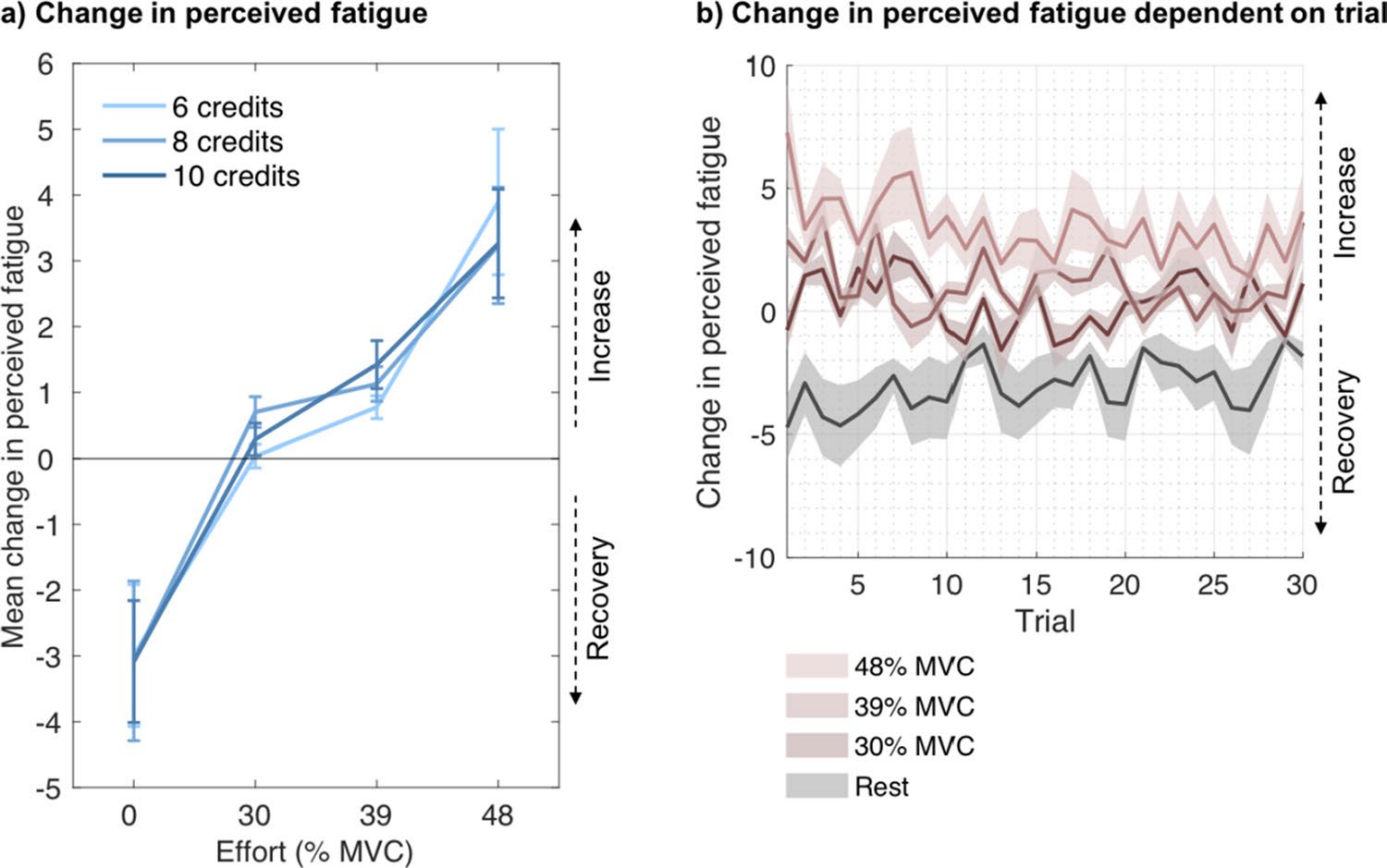
**Change in perceived fatigue in Experiment 3 as a function of effort level, reward, and trial number. (a)** Mean change in participants’ ratings on the question, “How tired do you feel?” from trial n-1 to trial n as a function of rest (0% MVC), effort level, and reward (credits earned). **(b)** Mean change in participants’ ratings on the question, “How tired do you feel?” from trial n-1 to trial n as a function of rest (0% MVC), effort level, and the trial in which the respective rest/effort level was required throughout the task. Only successful trials in which participants exerted the required amount of force and earned the credits associated with that trial were included. Error bars and shaded areas represent standard errors of the means.

In successful work trials (94.39% of those trials were successfully completed on average), higher effort was predictive of higher increases in fatigue (χ^2^(1) = 195.66, *p* <.001; Fig. 6a), and cumulative effort was associated with reduced increases in fatigue (cumulative effort: χ^2^(1) = 20.602, *p* <.001; cumulative effort × effort: χ^2^(1) = 6.000, *p* =.014; cumulative effort × reward: χ^2^(1) = 0.332, *p* =.564; cumulative effort × effort × reward: χ^2^(1) = 0.417, *p* =.519; Fig. 6b). However, the analyses did not reveal an effect of reward on changes in perceived fatigue (reward: χ^2^(1) = 0.548, *p* =.459; reward × effort: χ^2^(1) = 2.377, *p* =.123). Similar to Experiment 2, an additional LMM with force as outcome variable revealed that higher cumulative effort was associated with lower force production (χ^2^(1) = 69.645, *p* <.001).

In a separate analysis, including rest trials only, reward did not significantly affect recovery (χ^2^(1) = 0.141, *p* =.708), but participants recovered somewhat less with cumulative effort (χ^2^(1) = 14.553, *p* <.001). The interaction was not significant (χ^2^(1) = 0.018, *p* =.893). In sum, when the amount of reward was completely independent of the force exerted within a trial, moment-to-moment changes in perceived fatigue depended on the effort exerted, while there was no credible evidence that they depended on the reward obtained.

#### Recoverable and unrecoverable components underlie fluctuations in perceived fatigue

To verify the development of perceived fatigue resulting from repeated exertion and rest and to better understand its relation to perceived effort, we used computational modelling and tested whether similar recoverable and unrecoverable components would underlie fluctuations in the perception of fatigue in our two experiments.

**Fig. 7:**
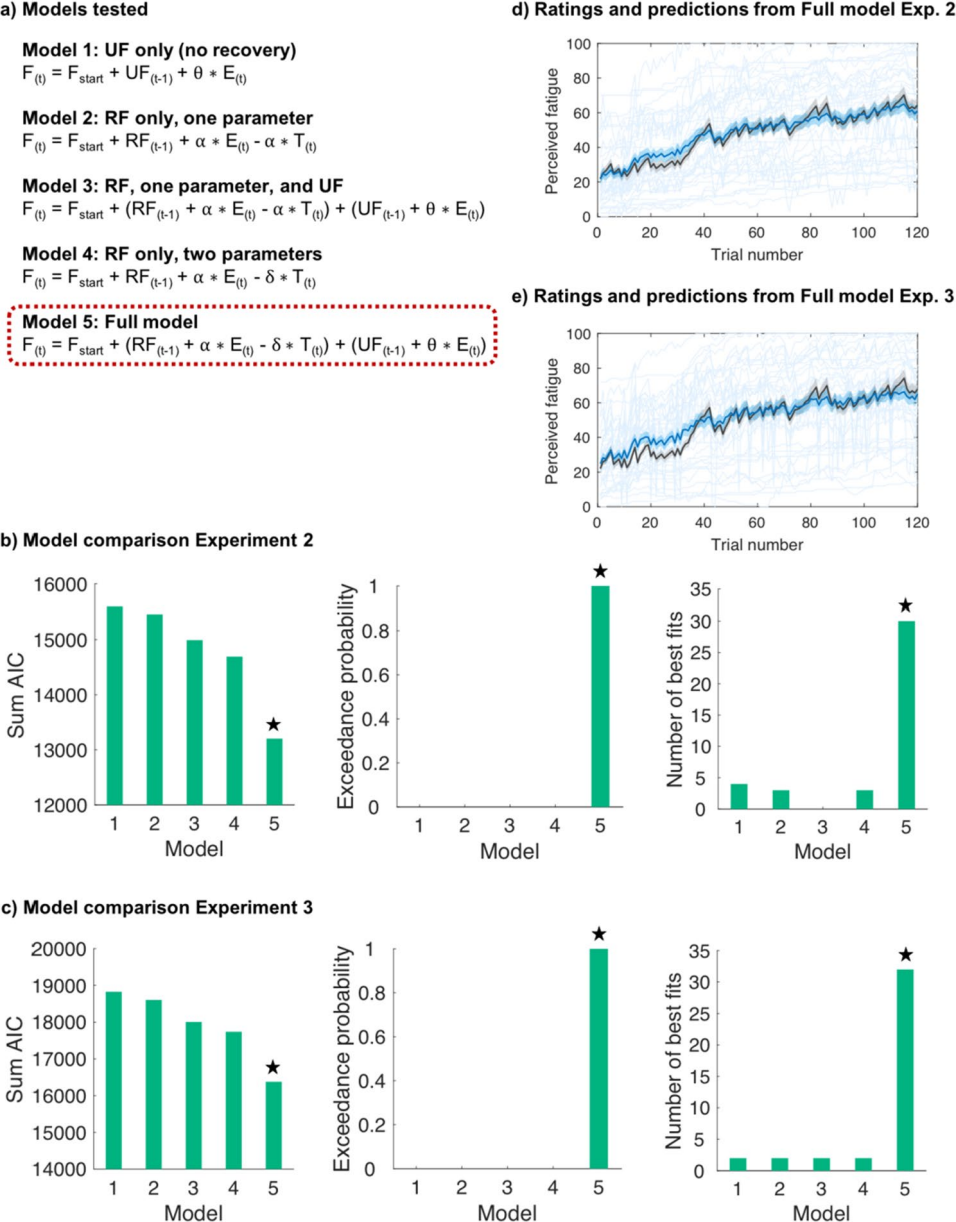
**Modelling perceived fatigue. (a)** List of all models compared. **(b)** Model comparison results for Experiment 2. **(c)** Model comparison results for Experiment 3. The x-axis is depicting the model number and the y-axis the sum of the Akaike Information Criterion (AIC) score, the Exceedance probability, or the number of participants for whom ratings were best explained by the respective model according to individual AICs, indicating that participants’ development of perceived fatigue was overall best described by the Full model (Model 5) in both experiments, as indicated by the star. **(d)** Development of perceived fatigue across the task in Experiment 2. **(e)** Development of perceived fatigue across the task in Experiment 3. Depicted are individual participants’ fatigue ratings (light blue) and group mean (blue) as a function of trial number, alongside mean fatigue ratings as predicted by the Full model (black). Shaded areas represent standard errors of the means. F_start_ = fatigue rating collected immediately before the first trial of the main task; F = fatigue on trial t; UF = unrecoverable fatigue; RF = recoverable fatigue; E = force exerted; T = time rested.

**Fig. 8:**
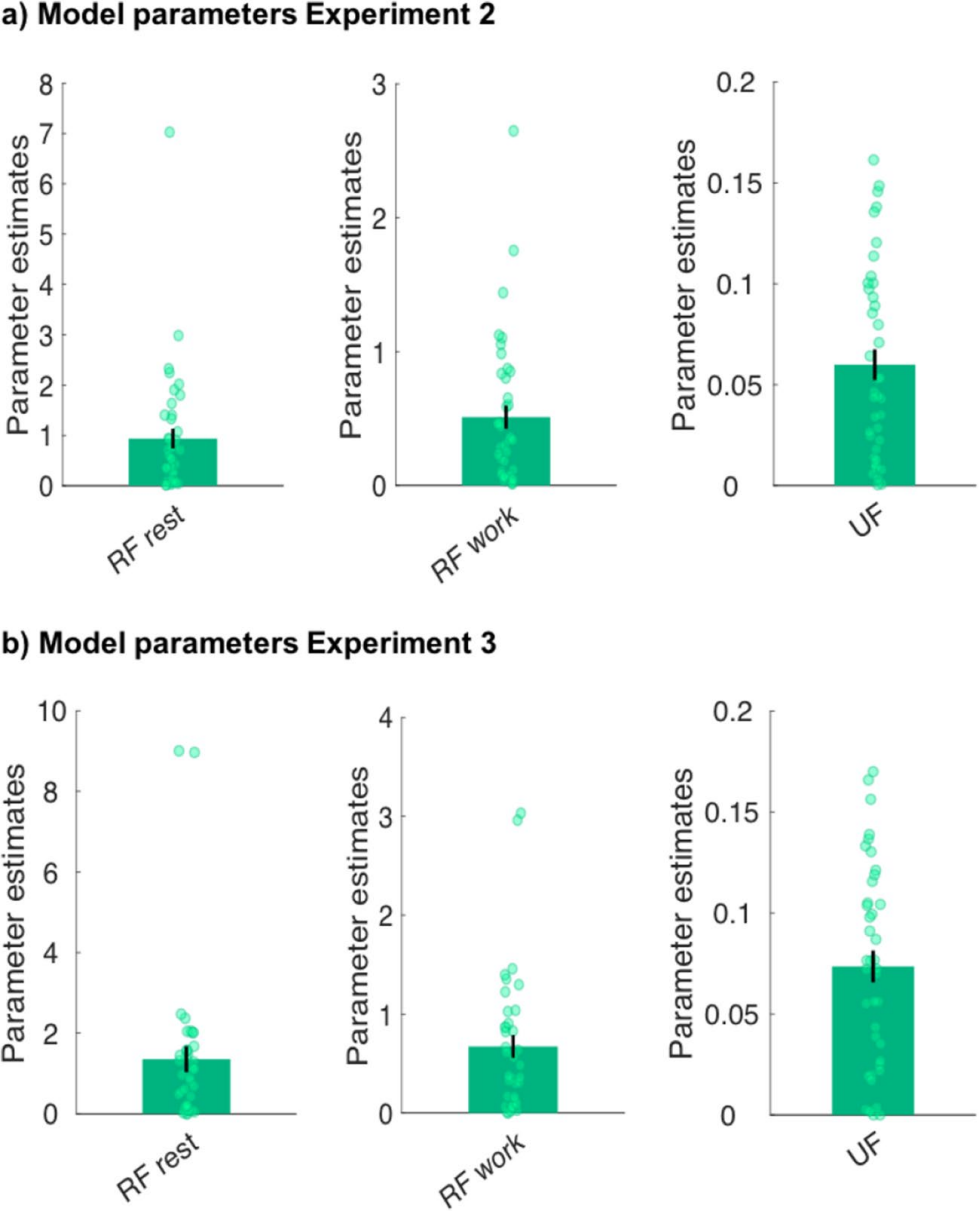
**Model parameters from the Full model in Experiments 2 and 3. (a)** Parameter estimates for each participant (green dot) and the average across participants for the two recoverable fatigue parameters (RF; *n* = 40) and the unrecoverable fatigue parameter (UF; *n* = 40) in Experiment 2. **(b)** Parameter estimates for each participant (green dot) and the average across participants for the two recoverable fatigue parameters (RF; *n* = 38) and the unrecoverable fatigue parameter (UF; *n* = 40) in Experiment 3. Two participants’ RF parameters were not included for display purposes due to high values. Error bars represent SEM.

Five different models were fitted to each participant’s ratings and compared by using AIC. Analyses revealed that the Full model, consisting of recoverable and unrecoverable fatigue components, best predicted participants’ perceived fatigue in both Experiments 2 and 3 in terms of both AIC and exceedance probability (Fig. 7). The winning model fit fatigue ratings well in both experiments (median *R*^2^ for Experiment 2 =.91; median *R*^2^ for Experiment 3 =.88; Figs. 7d and 7e). These findings highlight that the same model that can explain changes in sensations of effort over time can also account for changes in sensations of fatigue over trials. We additionally present parameter estimates for the winning model for both experiments (Fig. 8), as well as examples of participants with different parameter values (Supplementary Figs. 1b and 1c), to illustrate interindividual variability.

## Discussion

Theories of motivation have long posited that exerting effort leads to increases in sensations of fatigue, which in turn lead to higher perceptions of effort, reducing motivation (Boksem & Tops, 2008; Hockey, 2011; Müller & Apps, 2019). However, while many studies examine changes in performance (Asplund & Chee, 2013; Boksem et al., 2006; Dobryakova et al., 2013; Möckel et al., 2015; Wylie et al., 2017) or decisions to exert effort (Dobryakova et al., 2013; Massar et al., 2018; Müller et al., 2021; Müller & Apps, 2019) over time, very few have examined the dynamic, moment-to-moment nature of effort and fatigue sensations, nor offered a formal account computationally. Here, across three experiments using a grip force task, we show that people’s perception of effort and fatigue depend on the history of rests and the effort they have exerted. Furthermore, the same computational model, including recoverable and unrecoverable components of fatigue, can account for the dynamic changes in both perceptions of effort and fatigue, highlighting that the two processes may be closely interlinked as previous theories would predict. Additionally, we show that the effects of reward on perceptions of effort and fatigue may be driven by the fact that higher incentives increase levels of effort, meaning that a higher perception of effort is due to the increased exertion.

Our results demonstrate that predictions from existing theories of motivation (Hockey, 2011; Silvestrini et al., 2022) that were largely untested in terms of examining the dynamic, moment-to-moment nature of sensations of fatigue and especially effort, can be partially supported. Previous research had suggested that exerting effort increases fatigue, with higher efforts causing greater increases, and suggested that rests are restorative, reducing fatigue, and thus also sensations of effort (Matthews et al., 2023; Meyniel et al., 2014; Müller et al., 2021; Müller & Apps, 2019). Such predictions were borne out in the data. However, our results indicate that the effect of reward may only increase incentives for exerting more effort and thus increase perceptions of effort because people are working harder. This goes against previous accounts that suggested rewards can reduce sensations of effort or fatigue (Boksem et al., 2006; Boksem & Tops, 2008; Dobryakova et al., 2013; 2018). Moreover, whilst prior psychological and neuro-cognitive theories have provided useful insights into the effects of fatigue and the perception of effort, they were not formalised computationally.

By using computational modelling, it was possible to demonstrate that sensations of both fatigue and effort dynamically change with recoverable and unrecoverable components. Previously, this same model was put forward as a computational account for how motivation, and the willingness to exert effort, might be susceptible to fluctuating levels of fatigue (Matthews et al., 2023; Müller et al., 2021). Indeed, it has been shown to predict fluctuations in effort-based decisions both for physically and mentally effortful tasks and has been shown to be related to activity in frontostriatal systems (Matthews et al., 2023; Müller et al., 2021). However, the present results go beyond this to reveal that people actually perceive exerting the same amount of force as more effortful due to fluctuating recoverable and gradually increasing unrecoverable components of fatigue. Putting these findings together it suggests that on a moment-to-moment basis, fatigue fluctuates due to effortful exertion, which increases how effortful it is predicted the same task will be subsequently. Thus, when people can decide whether they are willing to expend effort for a reward, this increased perception of effort may lead them to treat it as more costly in their subjective evaluations. As a result, a higher reward may be needed for the effort to be pursued compared with before.

Previous work has often argued that fatigue may simply develop with time-on-task. Indeed, in a significant proportion of studies in cognitive psychology, trial number is included in analyses to control for the effects of fatigue. However, our results do not support the notion that time-on-task is a good proxy of fatigue. They demonstrate that fatigue develops on a momentary basis due to the efforts being exerted. Furthermore, rest trials, which were matched in terms of time to the effortful trials, do not lead to increases in fatigue. These differences can be attributed to advantages within our design. These include sampling subjective ratings at regular intervals, manipulating several levels of demand for effort in the task, and keeping effort required below each person’s capacity. This meant that we could measure changes in fatigue and effort perception as a function of very brief moments of exertion or rest. Deployment of such an approach may be useful for future experiments to uncover new insights into fatigue, effort, and motivation. It might be particularly interesting to use a similar approach during whole body exercise or during various cognitive tasks to examine similarities and potential differences and to more specifically test the contribution of brain systems, neurotransmitters, and peripheral factors to perceived effort and fatigue, as well as to differentiate for example ratings of executed exertion from ratings of perceived effort. For instance, theoretical accounts and empirical evidence suggested that the mechanisms and brain systems underlying fatigue in cognitive and physical tasks may be similar but partly distinct (Matthews et al., 2023; Müller et al., 2021; Müller & Apps, 2019). Eventually, in physical tasks at a certain intensity and certain duration, peripheral signals from the body and activity patterns in brain motor systems will likely have a more considerable impact on assessments of exertion, sensations of effort and fatigue, and associated drops in motivation and performance (Carroll et al., 2017; Hogan et al., 2020; Hu et al., 2022; Kuppuswamy, 2017; Nybo & Secher, 2004; Tanaka & Watanabe, 2012).

Previous research has also argued that rewards can reduce levels of fatigue or at least reduce the effects of fatigue on performance. This work has shown that when doing a demanding, fatiguing task, but then offering a significant reward, participants perform much better. This has been interpreted as fatigue having less of an impact (Boksem & Tops, 2008; Dobryakova et al., 2020). However, our results suggest that rewards increase sensations of fatigue or perceptions of effort when people know the incentive for their action but that they have no effect when they do not. How can these results regarding the relationship between rewards, perceived effort, and perceived fatigue be explained? Our results suggest that there are two factors at play, at least in the physical task presented here.

First, when higher rewards are at stake, and participants know this, they actually become more motivated, and increase the vigour of their actions, perhaps to increase their probability of success. This might appear to be a reduction in fatigue because performance at the task might improve. However, in fact this increases people’s sensations of fatigue and leads them to perceive things as more effortful. This is consistent with recent research having shown that rewards can modulate behaviour, such as a person’s vigour or accuracy, even over and above what would be required to obtain the rewards (Manohar et al., 2017; Oudiette et al., 2019; Shadmehr et al., 2019). This suggests that there can be a decoupling between people’s sensations of fatigue and their motivation when they are motivated to perform an action. Such findings therefore accord with recent evidence showing that people are sometimes very willing to exert very high effort when they are striving to achieve highly rewarding goals (Pisauro et al., 2024).

Second, when people do not know the incentive for an action, they cannot increase the vigour of their responses when there will be a higher reward and thus fatigue exclusively depends on the effort they exerted. This leads to them not becoming more fatigued after a higher reward. Crucially, in Experiment 3, participants were also made aware of the reward they had received, before providing their fatigue ratings, but this did not lead to a change in people’s fatigue rating. This provides compelling evidence that the receipt of a reward does not make people feel less fatigued and we speculate the same is true for the perception of effort, given the close links between these sensations in these experiments.

Heightened fatigue, and a reduced willingness to exert effort, are highly prevalent symptoms across many neurological and psychiatric conditions and can have some of the most debilitating effects (Bigliassi, 2015; Chaudhuri & Behan, 2004; Lerdal et al., 2009; Ryan et al., 2007; Wessely, 2001). To date, existing theoretical and empirical accounts of fatigue have typically treated fatigue as a static symptom. Although useful, this misses out the important information about how fatigue develops from moment-to-moment. That is, patients may differ in terms of how their symptoms develop during demanding tasks. A recent study, for example, showed that increased dopamine availability in Parkinson’s disease patients reduced their variability in physical exertion and was associated with increased accuracy of patients’ retrospective assessment of their amount of exertion (Padmanabhan et al., 2023). The computational model used in the present study, which quantifies each person’s sensitivity to the three parameters that define how the recoverable and unrecoverable components of fatigue develop, may prove useful to better understand the severe levels of fatigue in patient populations. Specifically, this work makes the case for undertaking research by using a computational psychiatry-based approach for understanding sensations of fatigue.

A limitation of our study is that a small proportion of participants did become significantly fatigued during the study to the extent where they rated themselves at the top of the rating scale. This is, of course, a common challenge when using rating scales in tasks such as this. Although our key results were not impacted by this effect, it does impair our ability to interpret some potentially interesting effects. Indeed, the linear mixed-effects models indicated that the effects of effort (and potentially rest) on ratings of fatigue slightly decreased over the course of the task. However, this would be the result one would expect if participants had reached the top of the scale, and especially when they slightly reduce their force exertion as they become fatigued. Thus, future work might profitably attempt to optimise these scales to test whether fatigue in fact increases the effect of each effort on subsequent fatigue ratings or has a reduced effect over trials.

## Conclusion

We show that the perception of effort and sensations of fatigue fluctuate dynamically on a moment-to-moment basis during physical exertion. A computational model of fatigue, previously validated on studies examining motivation, could capture the fluctuations in these subjective sensations. The findings demonstrate that fatigue and perceptions of effort are driven by two latent underlying components. There is an unrecoverable component which gradually increases over time with exerted effort. In addition, there is a recoverable component which increases with effort but declines with rest. These results demonstrate the computational dynamics of the perception of effort and fatigue, and highlight how closely interlinked they are.

## Supplementary Information

Below is the link to the electronic supplementary material.

Supplementary file1 (PDF 496 KB)

## Data Availability

Data are available on the Open Science Framework (OSF; https://osf.io/v2dzq/; Digital Object Identifier: 10.17605/OSF.IO/V2DZQ). The study was not preregistered.

## Code availability

Custom code is available on OSF (https://osf.io/sb9jf/; Digital Object Identifier: 10.17605/OSF.IO/SB9JF).
