# Supplementary material for: Computational signatures of exertion and rest underlie moment-to-moment dynamics of subjective perceptions of effort and fatigue
Source: Cogn Affect Behav Neurosci. 2026 Mar 9;26(2):650–68. doi: 10.3758/s13415-026-01417-1 (PMC13095941; doi:10.3758/s13415-026-01417-1)
Supplement: Supplementary file 1 — Supplementary file1 (PDF 496 KB) [file 13415_2026_1417_MOESM1_ESM.pdf]

Computational signatures of exertion and rest underlie moment-to-moment dynamics of subjective perceptions of effort and fatigue

Tanja Müller, Joseph Milton, Masud Husain, Matthew A. J. Apps

Supplementary Information

Supplementary Table 1

Coefficients and standardised coefficients with 95% confidence intervals (CIs) from the linear mixed-effects model on effort ratings in work trials in Experiment 1

Parameter	Coefficient	95% CI	Standardised coefficient	95% CI
intercept	12.67	[11.88, 13.47]	0.00	[-0.18, 0.18]
cumul_effort	0.64	[0.56, 0.72]	0.14	[0.12, 0.16]
effort	2.64	[2.56, 2.72]	0.59	[0.57, 0.61]
outcome	0.19	[0.11, 0.27]	0.04	[0.03, 0.06]
cumul_effort * effort	-0.02	[-0.10, 0.06]	0.00	[-0.02, 0.01]
cumul_effort * outcome	-0.06	[-0.14, 0.02]	-0.01	[-0.03, 0.00]
effort * outcome	0.09	[0.01, 0.17]	0.02	[0.00, 0.04]
cumul_effort * effort * outcome	0.05	[-0.03, 0.13]	0.01	[-0.01, 0.03]

Note. Predictors were coded as continuous variables. A subject-level random intercept was included.

Supplementary Table 2

Coefficients and standardised coefficients with 95% confidence intervals (CIs) from the linear mixed-effects model on effort ratings in rest trials in Experiment 1

Parameter	Coefficient	95% CI	Standardised coefficient	95% CI
intercept	0.48	[0.25, 0.71]	0.00	[-0.12, 0.12]
cumul_effort	-0.02	[-0.12, 0.07]	-0.01	[-0.06, 0.04]
outcome	0.02	[-0.08, 0.12]	0.01	[-0.04, 0.06]
cumul_effort * outcome	0.02	[-0.08, 0.12]	0.01	[-0.04, 0.06]

Note. Predictors were coded as continuous variables. A subject-level random intercept was included.

Supplementary Table 3

Coefficients and standardised coefficients with 95% confidence intervals (CIs) from the linear mixed-effects model on force in work trials in Experiment 1

Parameter	Coefficient	95% CI	Standardised coefficient	95% CI
intercept	0.74	[0.72, 0.75]	0.00	[-0.10, 0.10]
cumul_effort	0.00	[0.00, 0.01]	0.03	[0.02, 0.04]
effort	0.12	[0.12, 0.12]	0.87	[0.85, 0.88]
outcome	0.00	[0.00, 0.00]	0.01	[0.00, 0.02]
cumul_effort * effort	0.00	[0.00, 0.00]	-0.01	[-0.02, 0.00]
cumul_effort * outcome	0.00	[0.00, 0.00]	0.01	[-0.01, 0.02]
effort * outcome	0.00	[0.00, 0.00]	0.01	[0.00, 0.02]
cumul_effort * effort * outcome	0.00	[0.00, 0.00]	0.01	[-0.01, 0.02]

Note. Predictors were coded as continuous variables. A subject-level random intercept was included.

Supplementary Table 4

Coefficients and standardised coefficients with 95% confidence intervals (CIs) from the alternative linear mixed-effects model on force in work trials in Experiment 1

Parameter	Coefficient	95% CI	Standardised coefficient	95% CI
intercept	0.74	[0.72, 0.75]	0.00	[-0.11, 0.10]
cumul_effort	0.00	[0.00, 0.01]	0.02	[-0.02, 0.06]
effort	0.12	[0.11, 0.12]	0.86	[0.83, 0.90]
outcome	0.00	[0.00, 0.00]	0.01	[0.00, 0.02]
cumul_effort * effort	0.00	[0.00, 0.00]	-0.01	[-0.03, 0.01]
cumul_effort * outcome	0.00	[0.00, 0.00]	0.01	[0.00, 0.02]
effort * outcome	0.00	[0.00, 0.00]	0.01	[0.00, 0.02]
cumul_effort * effort * outcome	0.00	[0.00, 0.00]	0.01	[0.00, 0.02]

Note. Predictors were coded as continuous variables. A subject-level random intercept was included, and random slopes on the main effects of cumulative effort, effort and their interaction per participant were additionally included.

Supplementary Table 5

Coefficients and standardised coefficients with 95% confidence intervals (CIs) from the linear mixed-effects model on change in fatigue ratings in work trials in Experiment 2

Parameter	Coefficient	95% CI	Standardised coefficient	95% CI
intercept	1.18	[0.83, 1.54]	0.00	[-0.11, 0.10]
cumul_effort	-0.26	[-0.37, -0.16]	-0.08	[-0.11, -0.05]
effort	0.88	[0.78, 0.98]	0.26	[0.23, 0.29]
outcome	0.18	[0.08, 0.28]	0.05	[0.02, 0.08]
cumul_effort * effort	-0.11	[-0.21, -0.01]	-0.03	[-0.06, 0.00]
cumul_effort * outcome	-0.04	[-0.14, 0.06]	-0.01	[-0.04, 0.02]
effort * outcome	0.02	[-0.08, 0.12]	0.01	[-0.02, 0.04]
cumul_effort * effort * outcome	0.06	[-0.05, 0.16]	0.02	[-0.01, 0.05]

Note. Predictors were coded as continuous variables. A subject-level random intercept was included.

Supplementary Table 6

Coefficients and standardised coefficients with 95% confidence intervals (CIs) from the linear mixed-effects model on change in fatigue ratings in rest trials in Experiment 2

Parameter	Coefficient	95% CI	Standardised coefficient	95% CI
intercept	2.14	[1.10, 3.18]	0.00	[-0.26, 0.25]
cumul_effort	-0.13	[-0.26, 0.01]	-0.03	[-0.06, 0.00]
outcome	0.07	[-0.06, 0.20]	0.02	[-0.02, 0.05]
cumul_effort * outcome	0.02	[-0.11, 0.16]	0.01	[-0.03, 0.04]

Note. Predictors were coded as continuous variables. A subject-level random intercept was included.

Supplementary Table 7

Coefficients and standardised coefficients with 95% confidence intervals (CIs) from the linear mixed-effects model on force in work trials in Experiment 2

Parameter	Coefficient	95% CI	Standardised coefficient	95% CI
intercept	0.74	[0.73, 0.75]	-0.01	[-0.08, 0.07]
cumul_effort	-0.01	[-0.01, 0.00]	-0.04	[-0.05, -0.03]
effort	0.12	[0.12, 0.12]	0.89	[0.88, 0.90]
outcome	0.00	[0.00, 0.00]	0.01	[0.00, 0.02]
cumul_effort * effort	0.00	[0.00, 0.00]	-0.01	[-0.02, 0.00]
cumul_effort * outcome	0.00	[0.00, 0.00]	0.01	[0.00, 0.02]
effort * outcome	0.00	[0.00, 0.00]	0.01	[0.00, 0.02]
cumul_effort * effort * outcome	0.00	[0.00, 0.00]	-0.01	[-0.02, 0.00]

Note. Predictors were coded as continuous variables. A subject-level random intercept was included.

Supplementary Table 8

Coefficients and standardised coefficients with 95% confidence intervals (CIs) from the alternative linear mixed-effects model on force in work trials in Experiment 2

Parameter	Coefficient	95% CI	Standardised coefficient	95% CI
intercept	0.74	[0.73, 0.75]	-0.01	[-0.09, 0.06]
cumul_effort	-0.01	[-0.01, 0.00]	-0.05	[-0.08, -0.02]
effort	0.12	[0.12, 0.13]	0.89	[0.85, 0.92]
outcome	0.00	[0.00, 0.00]	0.01	[0.00, 0.02]
cumul_effort * effort	0.00	[0.00, 0.00]	-0.02	[-0.03, -0.01]
cumul_effort * outcome	0.00	[0.00, 0.00]	0.01	[0.00, 0.02]
effort * outcome	0.00	[0.00, 0.00]	0.01	[0.00, 0.02]
cumul_effort * effort * outcome	0.00	[0.00, 0.00]	-0.01	[-0.02, 0.00]

Note. Predictors were coded as continuous variables. A subject-level random intercept was included, and random slopes on the main effect of effort and on the main effect of cumulative effort per participant were additionally included.

Supplementary Table 9

Coefficients and standardised coefficients with 95% confidence intervals (CIs) from the linear mixed-effects model on change in fatigue ratings in work trials in Experiment 3

Parameter	Coefficient	95% CI	Standardised coefficient	95% CI
intercept	1.58	[0.85, 2.31]	0.00	[-0.13, 0.12]
cumul_effort	-0.40	[-0.58, -0.23]	-0.07	[-0.10, -0.04]
effort	1.25	[1.07, 1.42]	0.21	[0.18, 0.24]
outcome	0.07	[-0.11, 0.24]	0.01	[-0.02, 0.04]
cumul_effort * effort	-0.22	[-0.40, -0.04]	-0.04	[-0.07, -0.01]
cumul_effort * outcome	-0.05	[-0.23, 0.13]	-0.01	[-0.04, 0.02]
effort * outcome	-0.14	[-0.31, 0.04]	-0.02	[-0.05, 0.01]
cumul_effort * effort * outcome	-0.06	[-0.24, 0.12]	-0.01	[-0.04, 0.02]

Note. Predictors were coded as continuous variables. A subject-level random intercept was included.

Supplementary Table 10

Coefficients and standardised coefficients with 95% confidence intervals (CIs) from the linear mixed-effects model on force in work trials in Experiment 3

Parameter	Coefficient	95% CI	Standardised coefficient	95% CI
intercept	0.74	[0.73, 0.75]	-0.01	[-0.09, 0.08]
cumul_effort	-0.01	[-0.01, -0.01]	-0.06	[-0.07, -0.04]
effort	0.12	[0.12, 0.12]	0.87	[0.86, 0.88]
outcome	0.00	[0.00, 0.00]	0.00	[-0.02, 0.01]
cumul_effort * effort	0.00	[0.00, 0.00]	0.00	[-0.02, 0.01]
cumul_effort * outcome	0.00	[0.00, 0.00]	0.01	[0.00, 0.03]
effort * outcome	0.00	[0.00, 0.00]	0.00	[-0.01, 0.01]
cumul_effort * effort * outcome	0.00	[0.00, 0.00]	-0.01	[-0.02, 0.00]

Note. Predictors were coded as continuous variables. A subject-level random intercept was included.

Supplementary Table 11

Coefficients and standardised coefficients with 95% confidence intervals (CIs) from the linear mixed-effects model on change in fatigue ratings in rest trials in Experiment 3

Parameter	Coefficient	95% CI	Standardised coefficient	95% CI
intercept	3.05	[1.00, 5.10]	0.00	[-0.26, 0.26]
cumul_effort	-0.47	[-0.71, -0.22]	-0.06	[-0.09, -0.03]
outcome	0.05	[-0.20, 0.29]	0.01	[-0.02, 0.04]
cumul_effort * outcome	-0.02	[-0.26, 0.22]	0.00	[-0.03, 0.03]

Note. Predictors were coded as continuous variables. A subject-level random intercept was included.

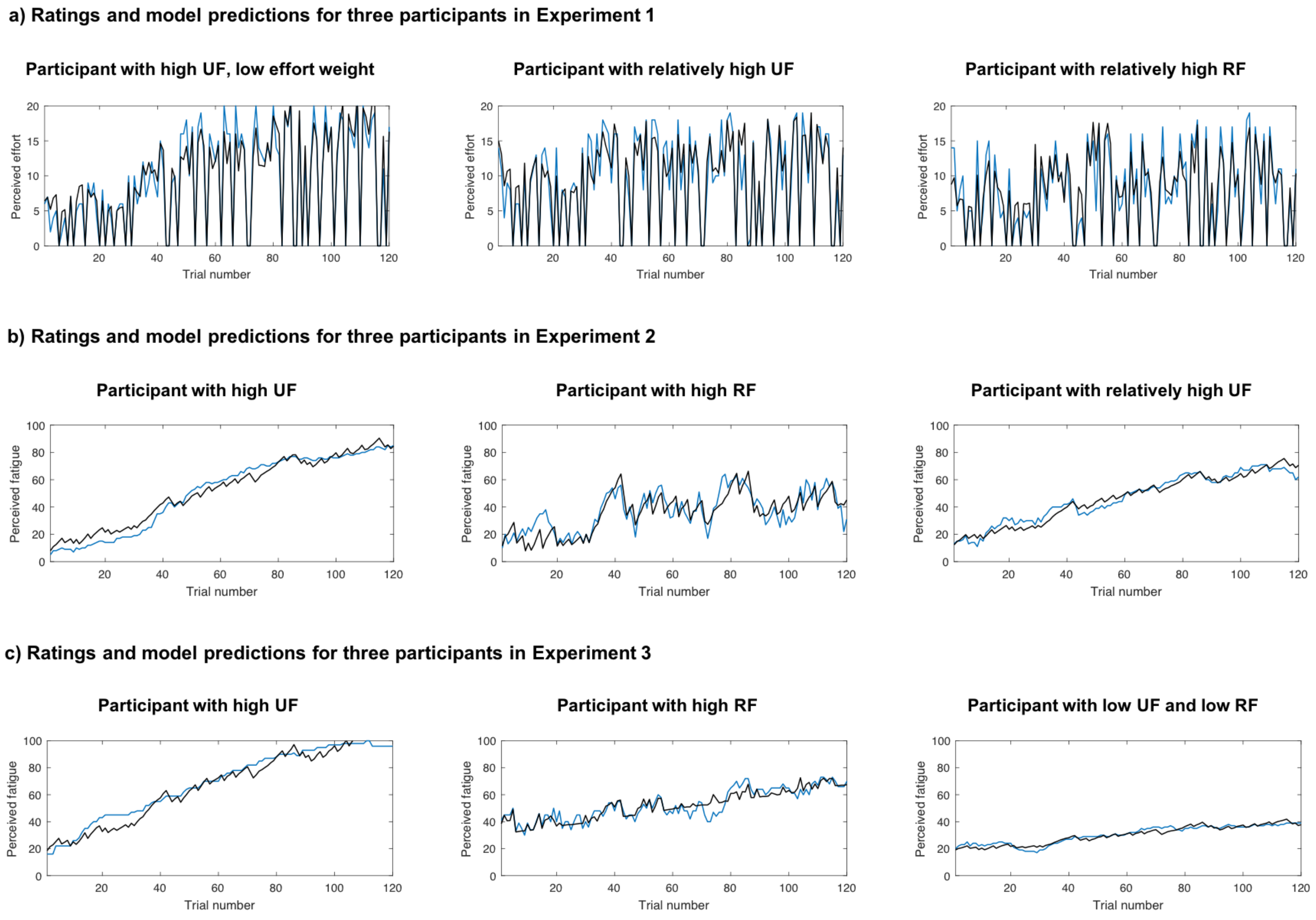

Supplementary Figure 1. Actual participant ratings (blue) and predicted ratings (black) from the best fitting models (full model) for three representative participants for Experiment 1 **(a)**, Experiment 2 **(b)** and Experiment 3 **(c)**. The plots illustrate how different model parameter values correspond to different actual and predicted perceived effort or fatigue developments. UF = Unrecoverable fatigue; RF = Recoverable fatigue.
